# Supplementary material for: Identification of novel PfEMP1 variants containing domain cassettes 11, 15 and 8 that mediate the Plasmodium falciparum virulence-associated rosetting phenotype
Source: PLoS Pathog. 2025 Jan 13;21(1):e1012434. doi: 10.1371/journal.ppat.1012434 (PMC11759366; doi:10.1371/journal.ppat.1012434)
Supplement: S2 Fig — SDS-PAGE of recombinant PfEMP1 domains. SDS-PAGE images of the recombinant PfEMP1 domains used to generate antibodies and/or used in erythrocyte binding assays and ELISAs. The his-tagged proteins were expressed in E. coli and purified by Ni-NTA or Co-NTA affinity chromatography, followed by size exclusion chromatography in some cases (this was done for all of the DBLα proteins except KE11VAR_R1 and PC0053-C.g410). Proteins IT4VAR60 DBLα, KE10VAR_R1 DBLα, PC0053VAR_R1 DBLα and PC0053-C.g96 DBLα had the his-tag removed by TEV cleavage as described previously (17, 48), whereas the other proteins did not. Gels were 4–12% Bis-Tris Novex gels run with MOPS or MES buffer at 200V for 55 minutes and stained with Instant Blue. Domain names are given below each image, except for KE10VAR_R1 domains which are lanes 1) Benchmark ladder 2) PFKE10VAR_R1 DBLα1.8 3) PFKE10VAR_R1 DBLγ7 4) PFKE10VAR_R1 DBLε11 5) PFKE10VAR_R1 DBLζ3 and 6) PFKE10VAR_R1 DBLε8. Molecular weight markers in kDa are shown for each gel. The NTS-DBLα preparations gave >90% of protein at the expected molecular weight (~50 kDa), whereas the other domain types had some degraded fragments and/or dimers. (DOCX) [file ppat.1012434.s002.docx]

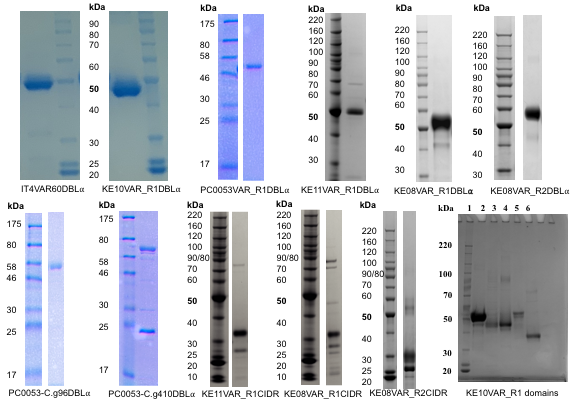


**Figure S2. SDS-PAGE of recombinant PfEMP1 domains.** SDS-PAGE images of the recombinant PfEMP1 domains used to generate antibodies and/or used in erythrocyte binding assays and ELISAs. The his-tagged proteins were expressed in *E. coli* and purified by Ni-NTA or Co-NTA affinity chromatography, followed by size exclusion chromatography in some cases (this was done for all of the DBLα proteins except KE11VAR_R1 and PC0053-C.g410). Proteins IT4VAR60DBLα, KE10VAR_R1DBLα, PC0053VAR_R1DBLα and PC0053-C.g96 had the his-tag removed by TEV cleavage as described previously (1, 2), whereas the other proteins did not. Gels were 4-12% Bis-Tris Novex gels run with MOPS or MES buffer at 200V for 55 minutes and stained with Instant Blue. Domain names are given below each image, except for KE10VAR_R1 domains which are lanes 1) Benchmark ladder, 2) PFKE10VAR_R1 DBLα1.8, 3) PFKE10VAR_R1 DBLγ7, 4) PFKE10VAR_R1 DBLε11, 5) PFKE10VAR_R1 DBLζ3 and 6) PFKE10VAR_R1 DBLε8. Molecular weight markers in kDa are shown for each gel. The NTS-DBLα preparations (except PC0053-C.g410) gave >90% of protein at the expected molecular weight (~50 kDa), whereas the other domain types had some degraded fragments and/or dimers.

**References:**

1. Ghumra A, Semblat JP, Ataide R, Kifude C, Adams Y, Claessens A, et al. Induction of strain-transcending antibodies against Group A PfEMP1 surface antigens from virulent malaria parasites. PLoS Pathog. 2012;8(4):e1002665.

2. Ghumra A, Khunrae P, Ataide R, Raza A, Rogerson SJ, Higgins MK, et al. Immunisation with recombinant PfEMP1 domains elicits functional rosette-inhibiting and phagocytosis-inducing antibodies to Plasmodium falciparum. PLoS One. 2011;6(1):e16414.
