# Supplementary material for: Identification of novel PfEMP1 variants containing domain cassettes 11, 15 and 8 that mediate the Plasmodium falciparum virulence-associated rosetting phenotype
Source: PLoS Pathog. 2025 Jan 13;21(1):e1012434. doi: 10.1371/journal.ppat.1012434 (PMC11759366; doi:10.1371/journal.ppat.1012434)
Supplement: S3 Fig — A) Forward and side scatter were used to gate on erythrocytes (RBC) and exclude debris. B) Mature pigmented-trophozoite- and schizont-infected erythrocytes (Mature IEs) were detected as the DNA/RNA high population by staining with 1/2500 dilution of Vybrant DyeCycle Violet (DNA stain) and 20μg/ml of ethidium bromide (DNA/RNA stain). C) PfEMP1 on the surface of live mature infected erythrocytes was detected with 20μg/ml of polyclonal rabbit IgG against NTS-DBLα (variant KE08VAR_R1 shown) followed by 1/1000 dilution of Alexa Fluor 647-conjugated goat anti-rabbit IgG secondary antibody (red). The negative control (blue) was the same parasite culture suspension stained with rabbit IgG against NTS-DBLα from an irrelevant PfEMP1 variant (HB3VAR03 shown) or non-immunised rabbit IgG. (DOCX) [file ppat.1012434.s003.docx]

**
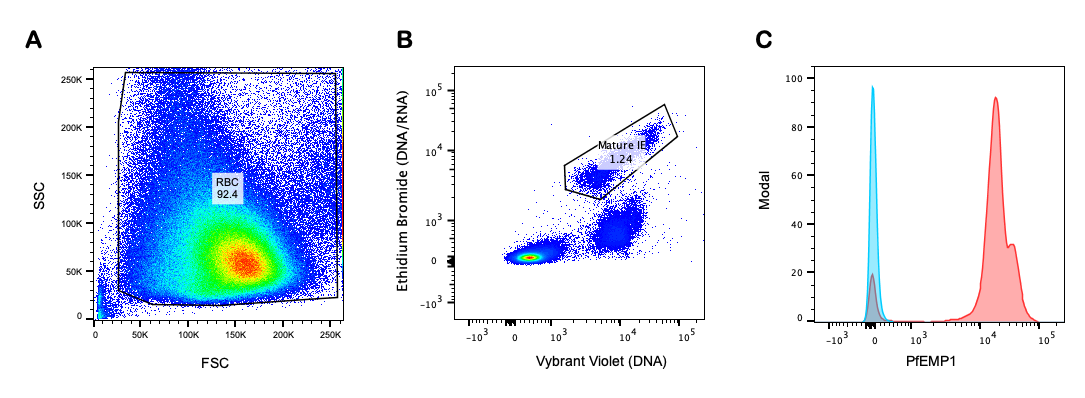
**

**Figure S3. Gating strategy to identify PfEMP1 on mature infected erythrocytes**. A) Forward and side scatter were used to gate on erythrocytes (RBC) and exclude debris. B) Mature pigmented-trophozoite- and schizont-infected erythrocytes (Mature IEs) were detected as the DNA/RNA high population by staining with 1/2500 dilution of Vybrant DyeCycle Violet (DNA stain) and 20μg/ml of ethidium bromide (DNA/RNA stain). C) PfEMP1 on the surface of live mature infected erythrocytes was detected with 20μg/ml of polyclonal rabbit IgG against NTS-DBLα (variant KE08VAR_R1 shown) followed by 1/1000 dilution of Alexa Fluor 647-conjugated goat anti-rabbit IgG secondary antibody (red). The negative control (blue) was the same parasite culture suspension stained with rabbit IgG against NTS-DBLα from an irrelevant PfEMP1 variant (HB3VAR03 shown) or non-immunised rabbit IgG.
