# Supplementary material for: Identification of novel PfEMP1 variants containing domain cassettes 11, 15 and 8 that mediate the Plasmodium falciparum virulence-associated rosetting phenotype
Source: PLoS Pathog. 2025 Jan 13;21(1):e1012434. doi: 10.1371/journal.ppat.1012434 (PMC11759366; doi:10.1371/journal.ppat.1012434)
Supplement: S4 Fig — Parasite cultures were incubated for 5 mins at 37°C with buffer only “Control” or with 5 μg/ml of trypsin, then stained with 20 μg/ml of PfEMP1 antibody (blue) or negative control rabbit IgG (red). A-B) PFKE08R+ with PFKE08VAR_R2 antibody; C-D) PFKE08R+ with PFKE08VAR_R1 antibody; E-F) PFKE10R+ with PFKE10VAR_R1 antibody. G-H) PC0053R+ with PC0053VAR_R1 antibody. One representative experiment out of two is shown. (DOCX) [file ppat.1012434.s004.docx]

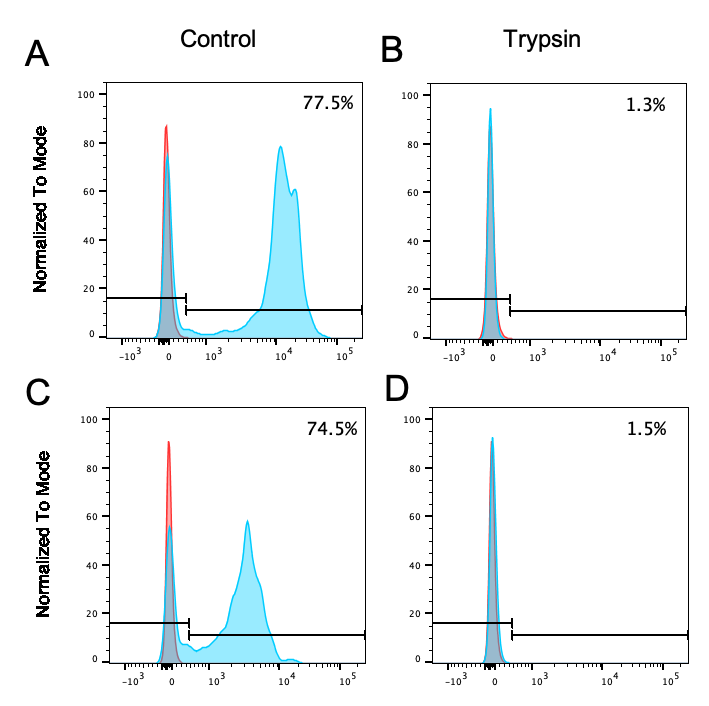


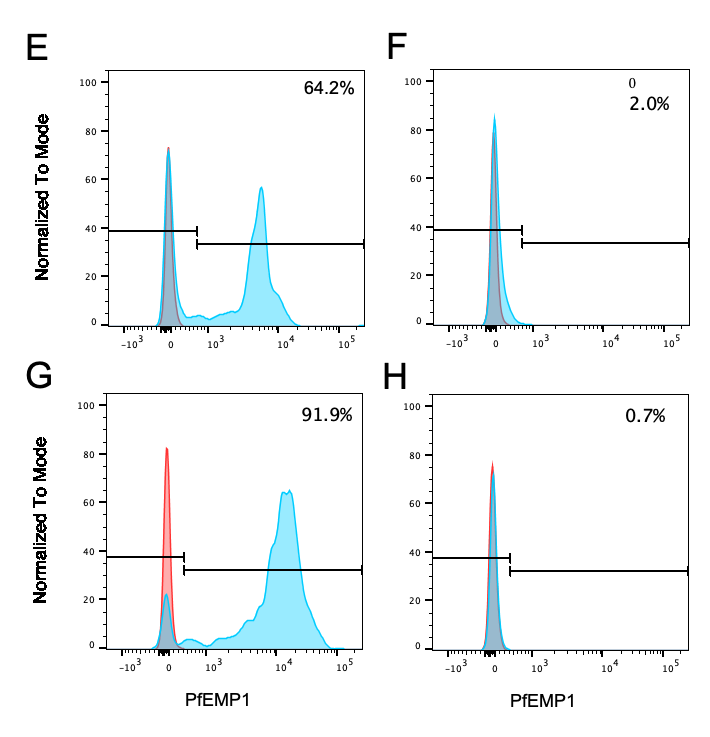


**Figure S4. Effect of low dose trypsinisation on PfEMP1 staining**. Parasite cultures were incubated for 5 mins at 37^o^C with buffer only “Control” or with 5 μg/ml of trypsin, then stained with 20 μg/ml of PfEMP1 antibody (blue) or negative control rabbit IgG (red). A-B) PFKE08R+ with PFKE08VAR_R2 antibody; C-D) PFKE08R+ with PFKE08VAR_R1 antibody; E-F) PFKE10R+ with PFKE10VAR_R1 antibody. G-H) PC0053R+ with PC0053VAR_R1 antibody. One representative experiment out of two is shown.
