# Supplementary material for: Identification of novel PfEMP1 variants containing domain cassettes 11, 15 and 8 that mediate the Plasmodium falciparum virulence-associated rosetting phenotype
Source: PLoS Pathog. 2025 Jan 13;21(1):e1012434. doi: 10.1371/journal.ppat.1012434 (PMC11759366; doi:10.1371/journal.ppat.1012434)
Supplement: S5 Fig — IQTREE was used to generate a maximum likelihood tree of the CIDRα1.5 amino acid sequences from the PFKE parasite line PfEMP1 repertoires, which had been aligned using MUSCLE. The amino acid domain boundaries were obtained from a previous study (27). Percentage bootstrap support is indicated on the nodes based on 1000 replicates, and the scale bar represents the number of changes per site. The CIDRα1.5b domains from the two rosetting variants are indicated in red (PFKE11.g448 = PFKE11VAR_R1 and PC0053-C.g687 = PC0053VAR_R1) and are found within a distinct clade within the tree supported by high bootstrap values. In this example, the tree is used as a way of visualising similarity between variants and is not intended to infer evolutionary descent. (DOCX) [file ppat.1012434.s005.docx]

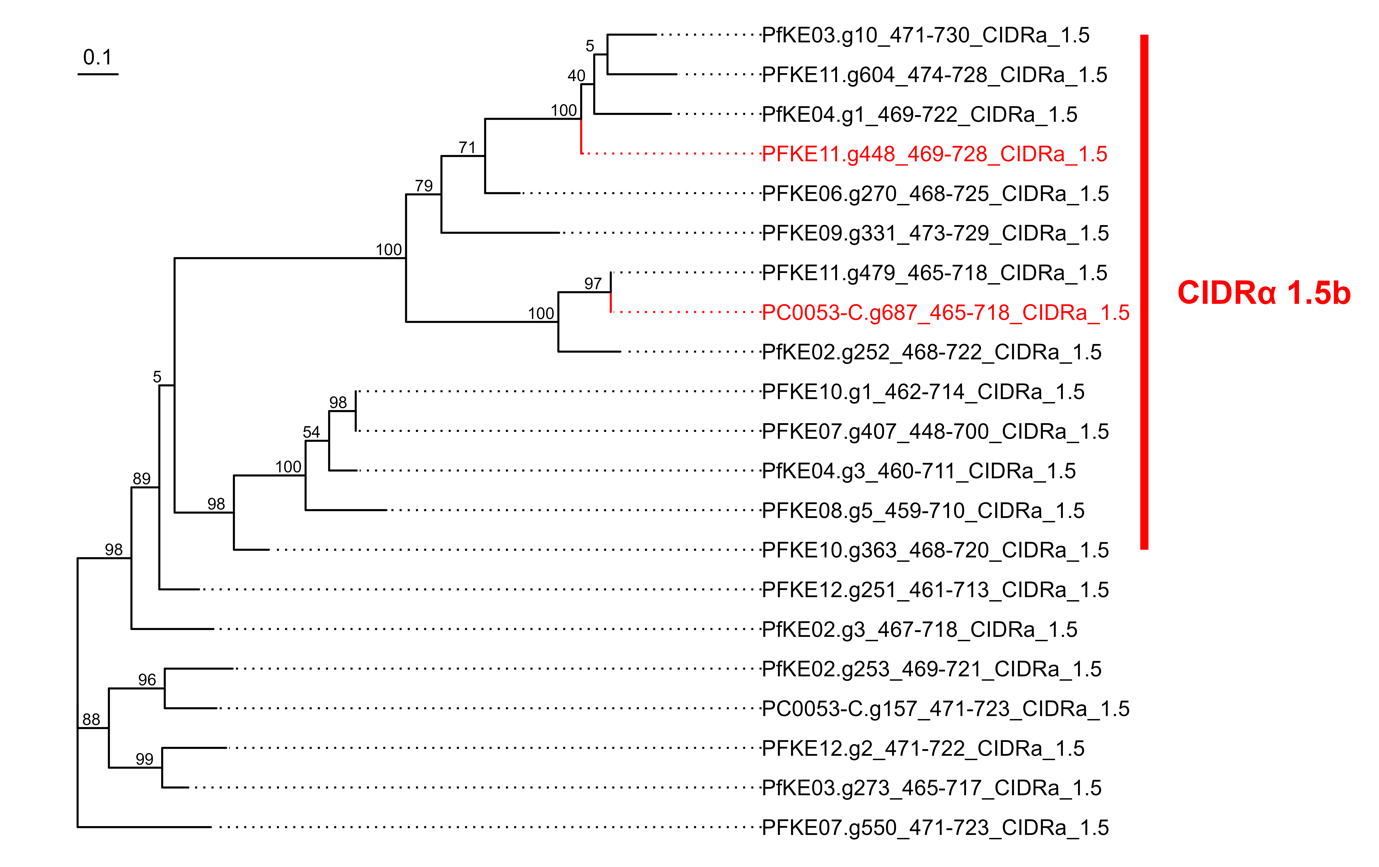


**Figure S5. Phylogenetic tree of CIDRα1.5 domains from the Kenyan parasite lines**. IQTREE was used to generate a maximum likelihood tree of the CIDRα1.5 amino acid sequences from the PFKE parasite line PfEMP1 repertoires, which had been aligned using MUSCLE. The amino acid domain boundaries were obtained from a previous study (1). Percentage bootstrap support is indicated on the nodes based on 1000 replicates, and the scale bar represents the number of changes per site. The CIDRα1.5b domains from the two rosetting variants are indicated in red (PFKE11.g448= PFKE11VAR_R1 and PC0053-C.g687 = PC0053VAR_R1) and are found within a distinct clade within the tree supported by high bootstrap values. In this example, the tree is used as a way of visualising similarity between variants and is not intended to infer evolutionary descent.

**Reference:**

1. Rask TS, Hansen DA, Theander TG, Gorm Pedersen A, Lavstsen T. *Plasmodium falciparum* erythrocyte membrane protein 1 diversity in seven genomes--divide and conquer. PLoS Comput Biol. 2010;6(9):e1000933.
