# Supplementary material for: Identification of novel PfEMP1 variants containing domain cassettes 11, 15 and 8 that mediate the Plasmodium falciparum virulence-associated rosetting phenotype
Source: PLoS Pathog. 2025 Jan 13;21(1):e1012434. doi: 10.1371/journal.ppat.1012434 (PMC11759366; doi:10.1371/journal.ppat.1012434)
Supplement: S1 Table — (PDF) [file ppat.1012434.s006.pdf]

Supplementary Table 1. ***var*** **gene profiling of the Kenyan parasite lines.**

Phenotype	Variant	Pie chart label	% *var* expression	5′ Ups group
**PFKE10/11019 R+ RF = 64%**				
Rosetting	*pfke10var_r1*	*pfke10var_r1*	82.6	A
Rosetting	*pfke10.g48*	a	4.3	B or C
Rosetting	*pfke10.g308*	b	8.7	Unknown
Rosetting	*pfke10.g50*	c	4.3	Unknown
**PFKE10/11019 R- RF = 0%**				
Control	*pfke10.g48*	a	15.0	B or C
Control	*pfke10.g237*	d	5.0	A
Control	*pfke10.g103*	e	5.0	B or C
Control	*pfke10.g15*	f	5.0	B or C
Control	*pfke10.g249*	g	5.0	B or C
Control	*pfke10.g181*	*g181*	20.0	B or C
Control	*pfke10.g473*	*g473*	15.0	B or C
Control	*pfke10.g283*	h	10.0	B or C
Control	*pfke10.g286*	i	5.0	B or C
Control	*pfke10.g524*	j	5.0	B or C
Control	*pfke10.g72*	k	5.0	B or C
Control	*px0203.g96*	l	5.0	Unknown
**PC0053/9197 R+ RF = 46%**				
Rosetting	*pc0053var_r1*	*pc0053var_r1*	50.0	A
Rosetting	*pc0053-c.g118*	a	6.2	B or C
Rosetting	*pc0053-c.g20*	*g20*	43.8	A
**PC0053/9197 R- RF <5%**				
Control	*pc0053-c.g111*	b	10.0	A
Control	*pc0053-c.g656*	c	10.0	B or C
Control	*pc0053-c.g667*	d	10.0	B or C
Control	*pc0053-c.g675*	e	10.0	B or C
Control	*pc0053-c.g684*	f	10.0	B or C
Control	*pc0053-c.g686*	g	10.0	B or C
Control	*pc0053-c.g20*	*g20*	20.0	A

Supplementary Table 1. ***var*** **gene profiling of the Kenyan parasite lines.**

Phenotype	Variant	Pie chart label	% *var* expression	5′ Ups group
Control	*pc0053-c.g268*	*g268*	20.0	B or C
**PFKE11/9775 R+ RF = 38%**				
Rosetting	*pfke11.g354*	*pfke11.g354*	13.0	B or C
Rosetting	*pfke11.g18*	*pfke11.g18*	13.0	B or C
Rosetting	*pfke11.g536*	*pfke11.g536*	17.4	A
Rosetting	*pfke11var_r1*	*pfke11var_r1*	17.4	A
Rosetting	*pfke11.g350*	a	4.3	A
Rosetting	*pfke11.g6*	b	4.3	A
Rosetting	*pfke11.g351*	c	4.3	B or C
Rosetting	*pfke11.g362*	d	4.3	B or C
Rosetting	*unknown 1*	e	4.3	Unknown
Rosetting	*unknown 2*	f	4.3	Unknown
Rosetting	*unknown 3*	g	4.3	Unknown
Rosetting	*unknown 4*	h	4.3	Unknown
Rosetting	*unknown 5*	i	4.3	Unknown
**PFKE11/9775 R- RF = 1%**				
Control	*pfke11.g140*	*pfke11.g140*	21.7	B or C
Control	*pfke11.g536*	*pfke11.g536*	13.0	A
Control	*pfke11.g671*	*pfke11.g671*	13.0	B or C
Control	*pfke11.g351*	c	4.3	B or C
Control	*pfke11.g137*	j	4.3	B or C
Control	*pfke11.g217*	k	4.3	B or C
Control	*pfke11.g295*	l	4.3	B or C
Control	*pfke11.g338*	m	4.3	B or C
Control	*pfke11.g352*	n	4.3	B or C
Control	*pfke11.g444*	o	4.3	B or C
Control	*pfke11.g480*	p	4.3	B or C
Control	*pfke11.g647*	q	4.3	B or C
Control	*unknown 6*	r	4.3	Unknown
Control	*unknown 7*	s	4.3	Unknown
Control	*unknown 8*	t	4.3	Unknown

Supplementary Table 1. ***var*** **gene profiling of the Kenyan parasite lines.**

Phenotype	Variant	Pie chart label	% *var* expression	5′ Ups group
**PFKE08/9605 R+ RF = 64%**				
Rosetting	*pfke08var_r1*	*pfke08var_r1*	29.2	A
Rosetting	*pfke08var_r2*	*pfke08var_r2*	62.5	B or C
Rosetting	*pfke08.g355*	a	4.2	A
Rosetting	*pfke08.g424*	b	4.2	B or C
**PFKE08/9605 R- RF = 1%**				
Control	*pfke08.g355*	a	26.3	A
Control	*pfke08var_r1*	c	5.3	A
Control	*pfke08.g228*	d	5.3	B or C
Control	*pfke08.g239*	e	5.3	B or C
Control	*pfke08.g250*	f	10.5	B or C
Control	*pfke08.g290*	g	5.3	B or C
Control	*pfke08.g356*	*g356*	15.8	B or C
Control	*pfke08.g393*	h	5.3	B or C
Control	*pfke08.g486*	i	5.3	B or C
Control	*unknown 1*	j	5.3	Unknown
Control	*unknown 2*	k	5.3	Unknown
Control	*unknown 3*	l	5.3	Unknown
