# Supplementary material for: Identification of novel PfEMP1 variants containing domain cassettes 11, 15 and 8 that mediate the Plasmodium falciparum virulence-associated rosetting phenotype
Source: PLoS Pathog. 2025 Jan 13;21(1):e1012434. doi: 10.1371/journal.ppat.1012434 (PMC11759366; doi:10.1371/journal.ppat.1012434)
Supplement: Attachment — Submitted filename: Reviewers comments-PLoS Pathogens.pdf [file ppat.1012434.s009.pdf]

The research article ‘Identification of novel PfEMP1 variants containing domain cassettes 11, 15 and 8 that mediate the Plasmodium falciparum virulence associated rosetting phenotype’ demonstrates the role of other DC cassettes in rosetting. The authors identified PfEMP1s expressed in kenyan field isolates and studied their role in rosetting. Importantly authors showed a new variant of DC cassette involved in rosetting where they have performed experiments to directly co-relate PfEMP1s with rosetting or cytoadherence phenomena. They also identified PfEMP1s expressed in field isolates and using antibody inhibition assays established their roles in rosetting. Their work also depicted that IgM binding of one of the isolates suggesting this phenotype is found among clinical isolates.

Major points:

1. The authors in the manuscript first discuss about field isolates and then role of TM180 var in rosetting. In my opinion the authors should discuss first about TM180 followed by their data on field isolates.
2. Although the authors mention that the work on the host receptor is for future studies. However inhibition experiment with heparan sulfate would provide an idea whether the rosetting is similar to other DC cassettes especially for the parasite that shows IgM binding phenotype.

Minor points:

1. The authors should perform spell check like ‘analyses’ in line 331 and ‘alos’ in line 356.
