# Supplementary material for: Identification of novel PfEMP1 variants containing domain cassettes 11, 15 and 8 that mediate the Plasmodium falciparum virulence-associated rosetting phenotype
Source: PLoS Pathog. 2025 Jan 13;21(1):e1012434. doi: 10.1371/journal.ppat.1012434 (PMC11759366; doi:10.1371/journal.ppat.1012434)
Supplement: Attachment — Submitted filename: Response to reviewers.docx [file ppat.1012434.s010.docx]

The concerns raised by the reviewers that need to be addressed are:

Major concerns
1. Experimental: The authors show by FACS that the KE08R+ line stained positively with two different PfEMP1 antibodies, indicates they are expressing two variants. This should be validated by IFA and show co-staining with two anti-PfEMP1 antibodies.

*This additional experiment has not been done because the antibodies to both variants are rabbit polyclonals, therefore dual staining by indirect IFA is not possible because both antibodies use the same secondary antibody. Dual staining could be done by directly conjugating each of the polyclonal antibodies with a different fluorophore, but this experiment is beyond the scope of the current manuscript, whose focus is to identify rosette-mediating PfEMP1 variants. We have inserted some extra discussion to indicate that the dual staining experiment should be done in future (lines 386-388 clean/502-504 tracked).*

Furthermore, the authors use trypinisation to demonstrate rosetting is mediated by PfEMP1, and FACS or western to demonstrate this loss of PfEMP1 is indeed responsible would validate this.

*Additional experiments have been done as described in the Results lines 210-212 clean/244-246 tracked and the Methods 537-542 clean/732-737 tracked and an extra flow cytometry figure (Figure S4) has been added. These experiments show that the predominant PfEMP1 variant in each of the new rosetting lines is cleaved by low dose trypsin treatment, consistent with the rosetting data.*

2. What is the frequency of known rosetting DBLa1.2/1.5/1.6/1.8 domains and the rosetting motifs within or outside cassettes. This information could be gleaned from the abundant var gene assemblies from the Pf3K project provided by Otto et al Wellcome Open Research 2019.

*The domain analysis has been done as described in the Results lines 294-302 clean/360-368 tracked and the Methods (619-632 clean/822-843 tracked) and an extra figure has been added (Figure 12). This shows that DBLa1.5/1.6/1.8 domains usually occur within their respective domain cassettes (DC16 and DC11), suggesting limited recombination with other var gene types. DBLa1.2 shows a different pattern, with only half of all DBLa1.2 domains occurring within DC15. Similar analysis of the motifs is challenging and gave ambiguous results due to their short length and redundancy, so we have not included that here.*

Minor concerns
1. The authors in the manuscript first discuss about field isolates and then role of TM180 var in rosetting. Suggestion is to revise the order and discuss first about TM180 followed by the data on field isolates.

*We thank the reviewer for this potentially useful suggestion. However, we tried this alternative structure, and in our view, it made the logical flow of the manuscript harder to follow. In addition, the main focus of the manuscript is the Kenyan parasite lines, and this is clearer when they are presented first. For these reasons, we have not revised the order, as to do so does not improve clarity.*

2. Fix references as indicated by Reviewer 3, including providing more current and accurate reviews for the model of monoallelic expression.

*The referencing has been corrected and updated as suggested using a recent review article reference 51 (Florini et al., 2022 Front Cell Dev Biol PMID: 35350381) lines 217 and 383 clean/251 and 499 tracked.*

3. The manuscript was poorly written in parts and contained many spelling mistakes that detracted from the manuscript. The manuscript needs proof reading to ensure these errors are fixed.

*We are sorry that the reviewer found the manuscript to be poorly written in parts. We have made minor edits to the text to increase readability throughout, and have proof-read and* *corrected* *any spelling mistakes.*

**Part II – Major Issues: Key Experiments Required for Acceptance**

Reviewer Comments (if any, and for reference):

Reviewer #1: 1. Since Authors have all required antibodies available it would be nice to see co-staining with two anti-PfEMP1 antibodies on the same parasite with KE08R+, where authors have shown expression of two PfEMP1.

*Response given above*.

2.Authors already have all required antibodies against PfEMP1s. It would be very nice to include a figure showing loss of PfEMP1 upon trypsinization using FACS/or western blot analysis.

*Response given above.*

Reviewer #2: 1.The authors in the manuscript first discuss about field isolates and then role of TM180 var in rosetting. In my opinion the authors should discuss first about TM180 followed by their data on field isolates.

*Response given above*

Although the authors mention that the work on the host receptor is for future studies. However inhibition experiment with heparan sulfate would provide an idea whether the rosetting is similar to other DC cassettes especially for the parasite that shows IgM binding phenotype

*The heparin sensitivity of the Kenyan rosetting lines has been added to the Results lines 172-173 clean/193-194 tracked, and the Methods lines 459-462 clean/598-614 tracked and Figure 2 parts C-E.*

Reviewer #3: The conserved domain cassette structure of var genes has featured a lot in attempts to associate sequences with disease and this makes sense as selection for adhesion seems the most probable reason for the conserved domain cassettes. In this light the information on DC15 is useful but could include a succinct exploration of the frequency of known rosetting DBLa1.2/1.5/1.6/1.8 domains and the rosetting motifs within or outside cassettes. There are abundant var gene assemblies from the Pf3K project provided by Otto et al Wellcome Open Research 2019 that could be used for this.

*Response given above.*

**Part III – Minor Issues: Editorial and Data Presentation Modifications**
Reviewer #1: Manuscript has a lot of sections poorly written and contains errors that should not be part of final draft. I highly encourage authors to go through the manuscript multiple times to remove those mistakes which otherwise restricts the flow for readers. I do understand these are benign mistakes but it is best if they could be minimised.

Reviewer #2: 1. The authors should perform spell check like ‘analyses’ in line 331 and ‘alos’ in line 356.

*The typo “alos” has been corrected. “Analyses” in line 331 was intended because more than one analysis was being referred to.*

Reviewer #3: The authors also identify a parasite that expresses two PfEMP1 simultaneously, in addition to the cited previous report of this phenomenon by Joergensen 2010 it has also been reported previously by Brolin 2009 Genome Biol, albeit the co-expressed genes were duplicated copies of var2csa. It is possible that dual expression is of biological relevance, it has however been shown by reverse genetics by Voss et al, as cited in the MS, but also by Dzikowski 2006 PLoS Pathog, that selection of a recombinant var gene silences other var genes. Although these experiments were all performed on the NF54-3D7 lineage, ItG has also been selected for monoallelic expression of A4 var in repeated publications by the Newbold lab, eg Kyes 2007 Mol Micro and for monoallelic expression of var2csa by multiple labs eg Duffy 2005 Mol Micro. I think the extent of experimental support for the model of monoallelic expression could be more clearly communicated, perhaps cite a current and accurate review? Although the cited Scherf paper was the first paper to propose monoallelic expression it proposes a disproven model of promiscuous ring stage expression and exclusive expression in trophozoites based on the expression of var1 that was subsequently shown to be atypical and constitutive in all parasites and is probably independent of allelic exclusive expression. I would recommend replacing this reference with a subsequent reference that accurately explains monoallelic expression as we understand it now, or at least supplementing it with a few such references to avoid readers drawing incorrect conclusions from the Scherf paper.

*As described above, we have replaced the Scherf paper with a recent review (Florini et al 2022) explaining current knowledge on monoallelic expression.*

Minor comments
Line 270 The cited paper from Lennartz et al shows that dblb12 does not bind icam1. DBLβ12 binds the host receptor gC1qR (Magallon Tejada plos pathog 2016),

*We are sorry for this error and have reworded the Results lines 275-277 clean/323-325 tracked to indicate the correct predicted binding phenotype for this domain as suggested by the reviewer.*

Line 336 Expression of DC11 was also shown to be upregulated in Papua in severe malaria by RNAseq in 2018 (tonkin-hill et al plos biol 2018)

*This reference has been added (reference 65).*

Fig 5 legend k keo8var -r3 should this read r2?

*This has been corrected.*
